# Assessing the Oxidative State of the Skin by Combining Classical Tape Stripping with ORAC Assay

**DOI:** 10.3390/ph15050520

**Published:** 2022-04-23

**Authors:** Reem M. Alnemari, Jana Brüßler, Cornelia M. Keck

**Affiliations:** Department of Pharmaceutics and Biopharmaceutics, Philipps-University of Marburg, Robert-Koch-Str. 4, 35037 Marburg, Germany; ph.dreem@hotmail.com (R.M.A.); jana_bruessler@web.de (J.B.)

**Keywords:** skin, oxidative state, ORAC, tape stripping, topical antioxidants, ascorbic acid, CoQ10

## Abstract

The antioxidant barrier system of the skin acts as the main defence against environmental pro-oxidants. Impaired skin oxidative state is linked to unhealthy conditions such as skin autoimmune diseases and cancer. Thus, the evaluation of the overall oxidative state of the skin plays a key role in further understanding and prevention of these disorders. This study aims to present a novel ex vivo model to evaluate the skin oxidative state by the measurement of its antioxidant capacity (AOC). For this the ORAC assay was combined with classical tape stripping and infrared densitometry to evaluate the oxidative state of the stratum corneum (SC). Outcomes implied the suitability of the used model to determine the intrinsic antioxidant capacity (iAOC) of the skin. The average iAOC of untreated skin was determined as 140 ± 7.4 µM TE. Skin exposure to UV light for 1 h reduced the iAOC by about 17%, and exposure for 2 h decreased the iAOC by about 30%. Treatment with ascorbic acid (AA) increased the iAOC in a dose-dependent manner and reached an almost two-fold iAOC when 20% AA solution was applied on the skin. The application of coenzyme Q10 resulted in an increase in the iAOC at low doses but decreased the iAOC when doses > 1% were applied on the skin. The results show that the combination of classical tape stripping and ORAC assay is a cost-effective and versatile method to evaluate the skin oxidative state and the pro-oxidate and antioxidative effects of topical skin treatments on the iAOC of the skin. Therefore, the model can be considered to be a valuable tool in skin research.

## 1. Introduction

The stratum corneum (SC) is the outer skin layer directly exposed to the external world and confronted with ultraviolet radiation (UV radiation), air pollutants, microorganisms and chemical pro-oxidants. It has a remarkable anatomy composed of corneocytes immersed in a lipid matrix resembling a brick-and-mortar structure as an essential part to protect the other organs and prevent epidermal water evaporation. It also includes a complex system of biochemicals to deal with imbalances caused by environmental pro-oxidants. This system is often referred to as the epidermal antioxidant barrier, comprising an endogenous antioxidant network [1,2]. The substances in this network can be divided mainly into small molecules and enzymatic antioxidants [3].

Small-molecule antioxidants prevent the formation of reactive oxygen species (ROS) and also act as free radical scavenging agents. They are distributed in the skin according to their solubility. For example, lipophilic antioxidants are embedded mainly in the cell membrane and lipid matrix, while hydrophilics are dispersed in the extracellular space [4,5]. They include L-ascorbic acid (AA), uric acid, glutathione, vitamin E, coenzyme Q10 (CoQ10) and carotenoids.

In contrast, enzymatic antioxidants are commonly described as detoxification enzymes and contain a cofactor that is primarily active by the electron transfer reaction [4]. The main antioxidants in this category are catalase, superoxide dismutase, glutathione peroxidase, thioredoxin and NAD(P)H:quinone reductase [6,7,8,9,10,11]. Furthermore, there are other biomolecules contributing to the antioxidant skin barrier with significant antioxidant activity, such as bilirubin, melanin, ferritin, L-carnitine and α-lipoic acid [4,12,13,14,15].

The epidermal antioxidant barrier has a crucial role in maintaining the skin integrity, as the accumulation of free radicals in this organ leads to lipid and protein malformation. The prolonged malformation will result in protein carbonylation, lipid peroxidation, cytokine secretions and DNA modification. Eventually, the structure and function of the skin will be damaged, resulting in oxidative stress. This process has been proven to be the underlying cause of various skin diseases, such as atopic dermatitis, psoriasis, acne, vitiligo and skin cancer [16,17,18,19].

There are several topical approaches to support the epidermal antioxidant barrier. Most important is the use of sunscreens and antioxidants. As a matter of fact, antioxidants are essential, even in most sunscreen formulations, because some UV filters are generating free radicals when they are exposed to UV radiation [20]. AA, tocopherols and CoQ10 are among the most often used antioxidants with extensive studies on their benefits [21,22,23]. In addition to that, carotenoids and polyphenols are also available in the market as cosmetics of natural origin, with rising popularity. Antioxidants application provides a direct source to the skin, in which they have a vital role to provide photoprotection, free radicals scavenging and anti-inflammatory action as well as maintaining the normal functions and physiology. Moreover, they enhance the skin’s ability to defend oxidative stress and thus can reduce skin aging at the cellular level [24].

Further investigations and understanding of skin oxidative state are a demanding need today. As the SC is the front line of the epidermal antioxidant barrier, it must be the starting point in this research area. Various techniques are already utilized to examine it, including invasive and noninvasive methods. Tape stripping is used most often, with the advantages of the simplicity and efficiency of layer by layer SC removal, enabling the further noninvasive ex vivo study of its elements [25,26].

Measuring the oxidative state of the skin using tape stripping or other skin investigation techniques has been studied previously. Examples are the investigation of carbonylated protein by Fujita et al. [27], the free radicals studies via Electron Spin Resonance (ESR) spectroscopy [28,29] and the use of ultraweak photon emission (UPE) in human and porcine skin [30,31]. In addition, it is possible by the detection of the skin oxidative markers, such as the peroxidised lipids (MDA and 4-HNE) [32], the catalase as antioxidant enzymes [33] and the cholinergic system response (mainly acetyl choline and acetylcholine esterase) which was associated with vitiligo pathogenesis [34].

Nevertheless, the available approaches are complicated, costly, not suitable for routine check-ups and require specialised devices. Hence, a simpler novel ex vivo method is presented in this paper to assess skin oxidative state, in which the ORAC (Oxygen Radical Absorbance Capacity) assay and tape stripping are combined. Today, many different assays are available for the determination of the antioxidant capacity. However, from all the tests available, the ORAC assay is considered to be a highly sensitive and holistic assay, because it provides a direct measure of the hydrophilic and lipophilic AOC at the same time, whereas many other assays only provide the hydrophilic or lipophilic AOC. In addition, ORAC can be used for both slow- and fast-reacting antioxidants. Due to these advantages, the ORAC assay was selected for this study [35]. Porcine skin was chosen, as literature screening indicated that porcine skin is a successful candidate to surrogate human skin, with marked similarities in skin histology and physiology [36,37,38]. The use of the ex vivo porcine ear model allows the use of skin that is still connected to the cartilage. Hence, the skin is in its physiological tension and condition, and no artifacts, i.e., disrupted skin barrier, squeezed skin with water on the skin surface, etc., are created during the preparation [39,40]. Thus, those methods were used and combined, and a specific term was applied to describe the ability of porcine skin’s antioxidant system to scavenge free radicals: intrinsic antioxidant capacity (iAOC). Using this term as the main concept, the first aim of this study was to define the normal range and the effects of several conditions on the iAOC of porcine skin. Therefore, three fundamental parts were established to test the model’s ability and reliability to fulfil this aim. The second aim was to expand the application of the proposed model via an additional fourth study.

**Figure 1:**
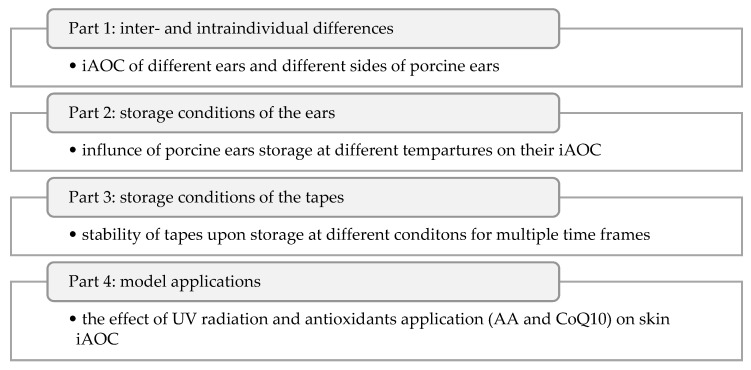
Study outline.

In the first part, the iAOC from different ears (interindividual) and within the same ear (intraindividual) was studied. In the second and third parts, the impact of storage conditions of ears and tapes on the iAOC were investigated. After that, the final part represents the model applications by studying the treatment impact of UV radiation and topical antioxidants on skin iAOC. The study outline is illustrated in Figure 1.

## 2. Results

### 2.1. ORAC Optimisation and Validation

**Figure 2:**
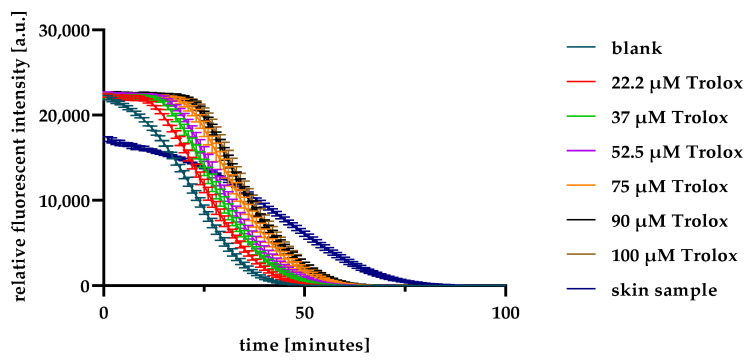
ORAC assay decay curves for Trolox and skin sample.

The specificity of ORAC assay for the antioxidants is already proven in the literature [41]. Accordingly, the assay was selected and optimised for the device and chemicals with final concentrations of 125 mM for AAPH and 1 µM for fluorescein. The prepared Trolox concentrations succeed to complete the course of the reaction and resulted in a linear relationship with the net area under the curve (AUC). The following step was to adjust the reaction with the skin samples, i.e., the SC extract obtained after tape stripping. Thus, the assay was applied using preliminary samples, and results showed that the reaction required approximately 100 min to achieve the full decay curve once the skin samples were added, as shown in Figure 2. This time frame was longer than the required time to achieve a full curve decay of Trolox samples because skin samples contained several antioxidants with different reaction times (fast- and slow-reacting) for each of them.

Afterwards, the assay was subjected to validation for five independent runs. A relative standard deviation (%RSD) of <10% ensured a high precision of the method. Accuracy was defined as relative error (% RE) and was 93% to 104% within each batch. Additionally, the average correlation coefficient of calibration curves was 0.99 ± 0.004.

### 2.2. Inter- and Intraindividual Differences

**Figure 3:**
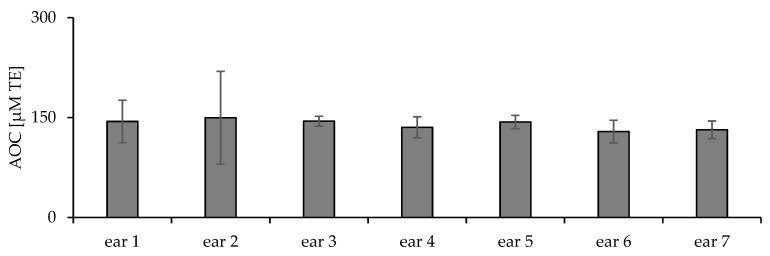
iAOC of different ears.

In the first part of the study, the variability of iAOC was investigated using skin from different ears (interindividual). Figure 3 shows the average iAOC obtained for seven different ears examined at six areas per ear, which ranged from 116 to 144 µM TE. There was no statistical difference between the average iAOC of the tested ears.

**Figure 4:**
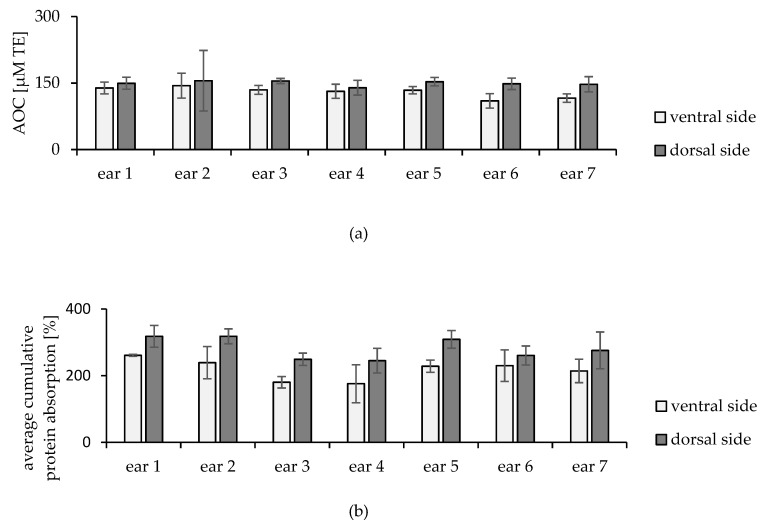
(**a**) iAOC (µM TE) and (**b**) average cumulative protein absorption (%) for dorsal and ventral sides of different ears, *p* = 0.0148 and 0.0016, respectively.

Additionally, detailed assessments were performed for the iAOC within the same ear (intraindividual). The iAOC for both sides of each ear (dorsal and ventral side) were assessed separately. Interestingly, a significantly higher iAOC was observed for the dorsal sides, as illustrated in Figure 4a (*p* = 0.0016).

To investigate the nature and the origin of these differences in the iAOC of the two sides, further analysis was necessary. Despite the accuracy of the implemented tape stripping protocol, one of the main anticipated factors was the quantity of removed skin layers. Therefore, the infrared densitometer (IR-D) measurements were examined, and the results reflected that more corneocytes were removed from the dorsal side than from the ventral side, as shown in Figure 4b (*p* = 0.0016).

Furthermore, the correlation between the average cumulative protein absorption and its corresponding iAOC was calculated for all the tested samples (42 areas). The analysis revealed a statistically significant positive correlation with a Pearson’s r of 0.49 (*p* = 0.0011).

**Figure 5:**
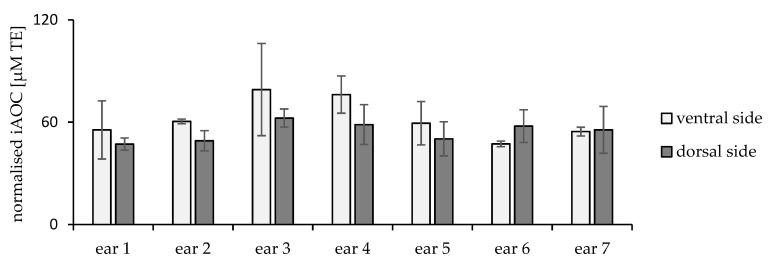
Normalised skin iAOC for dorsal and ventral ear sides.

Accordingly, the normalised iAOC was calculated and showed elevated values of the ventral side in most samples (Figure 5).

### 2.3. Storage Conditions of the Ears

**Figure 6:**
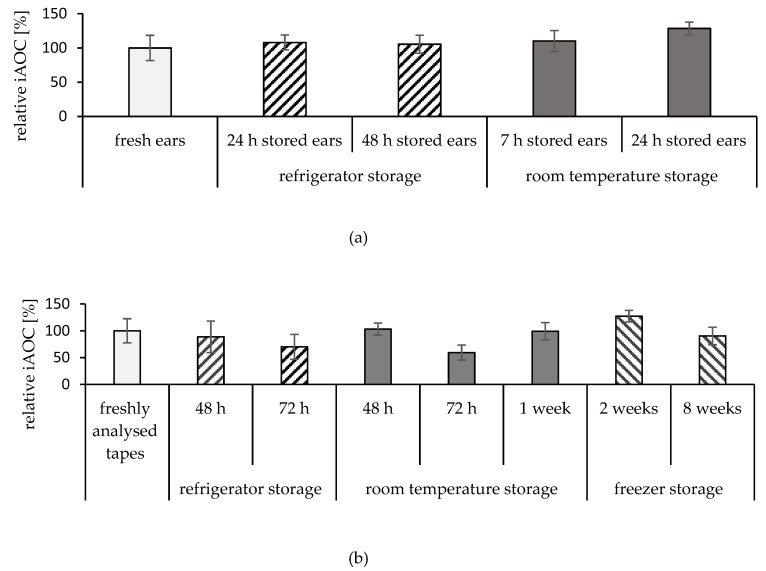
Influence of storage under different conditions on iAOC; (**a**) ear storage up to 48 h and (**b**) tapes storage up to 8 weeks.

As a next step, the effect of ears storage conditions on the iAOC of the skin was investigated. The results are expressed as the relative iAOC in comparison to the iAOC of freshly analysed samples. Storage in the refrigerator for up to 48 h did not influence the iAOC, as presented in Figure 6a. On the other hand, storage at room temperature for 24 h significantly enhanced the iAOC, up to 129% (*p* < 0.0001).

### 2.4. Storage Conditions of the Tapes

The impact of different storage conditions on the stability of the antioxidants in tapes after tape stripping was investigated in the next step. The results are shown in Figure 6b as % change of the iAOC compared to freshly analysed samples.

Only small changes in the iAOC were detected after tapes storage in the refrigerator and at room temperature for up to 48 h (89% and 103%, respectively). After that, a change occurred for samples that were stored for 72 h. The iAOC was reduced to 70% for the samples stored in refrigerator and to 59% for those stored at room temperature. Longer storage at room temperature for a week enhanced the iAOC to almost the same level as the freshly analysed samples (99%). In longer-term studies (up to eight weeks) at −20 °C frozen tapes showed no significant changes (Figure 6).

### 2.5. Model Application

#### 2.5.1. Effect of the UV Radiation on the Skin

**Figure 7:**
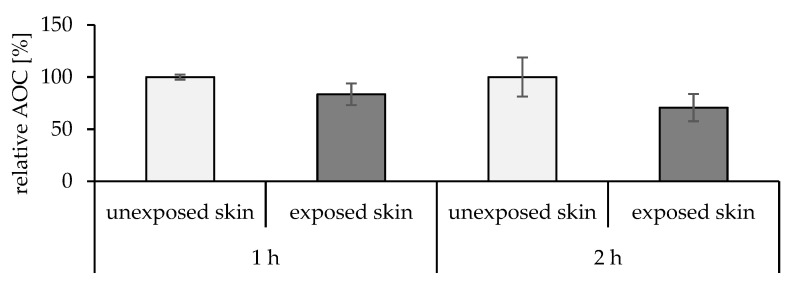
Effect of UV radiation on skin relative AOC for 1 h (*p* = 0.0011) and 2 h (*p* = 0.0064) compared to unexposed skin.

As solar radiation has a well-known pro-oxidative effect on skin and acts as an underlying factor for multiple skin conditions, it was crucial to initially assess our model considering its effect. UV lamp was used as radiation source for 1 and 2 h. The results revealed highly significant findings, as the exposed skin had a lower relative AOC by about 17% after 1 h and 30% after 2 h compared to the unexposed skin (Figure 7).

#### 2.5.2. Effect of Cosmeceuticals on Skin

##### In Vitro Studies

All the prepared antioxidants solutions were subjected to the in vitro AOC studies using ORAC assays.

**Figure 8:**
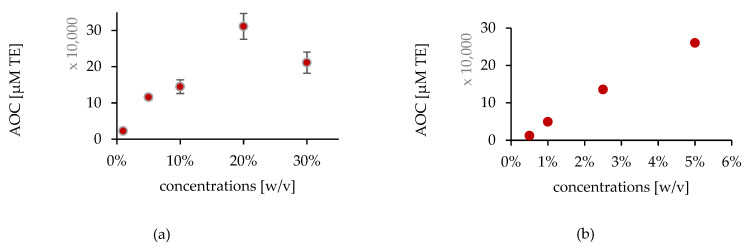
The in vitro AOC values using ORAC assay (**a**) for AA solutions (1–30% (w/v)), and (**b**) CoQ10 solutions (0.5–5% (w/v)).

Using ORAC, both agents showed a linear AOC with increased concentration, except for the 30% solution of AA, which seemed to have undergone a pro-oxidative reaction due to its high reactivity and concentration. The obtained linear regression (r) was 0.98 for AA solutions (1–20%) and 0.99 for CoQ10 solutions (0.5–5%). Results are illustrated in Figure 8.

##### Ex Vivo Studies

As the ability of our model to detect the changes in the skin oxidative state was successfully demonstrated, the model implantation was extended in the next step. Hence, the model was utilized to determine changes in skin AOC after topical application of antioxidants to measure the effect of these topical antioxidants on and in the skin.

**Figure 9:**
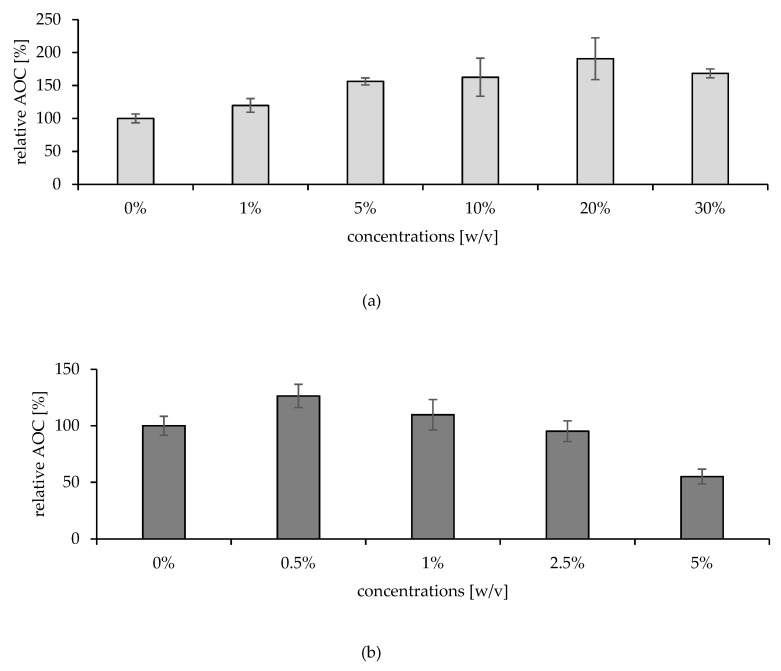
Relative AOC of the treated skin with (**a**) 1–30% (w/v) AA solutions (*p* = 0.0443), and (**b**) 0.5–5% (w/v) CoQ10 (*p* = 0.0001), compared to untreated skin.

For each ORAC analysis, the AOC of the removed SC (30 layers with classical tape stripping method) after treatment with antioxidants yielded a significant change. Interestingly, both active agents changed it in a concentration-dependent manner, as illustrated in Figure 9. All AA solutions improved skin AOC linearly (r = 0.97), except the 30% formulation. The achieved relative AOC was 120%, 156%, 162%, 190% and 168% for the formulations 1%, 5%, 10%, 20% and 30%, respectively.

In contrast, the impact of CoQ10 was variable but also strongly correlated to the applied dose. The lowest dose used was 0.5%, and it was able to improve the skin AOC by 26% (*p* = 0.0439). After that, the treatment with the higher doses started to trigger a negative impact, wherein the 1% resulted in a relative AOC of 110% and the 2.5% in 95%. Then, eventually, half of the skin AOC was consumed with the 5% application (*p* = 0.0012).

**Figure 10:**
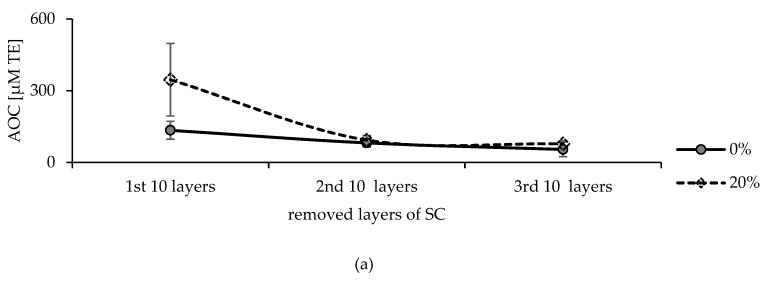

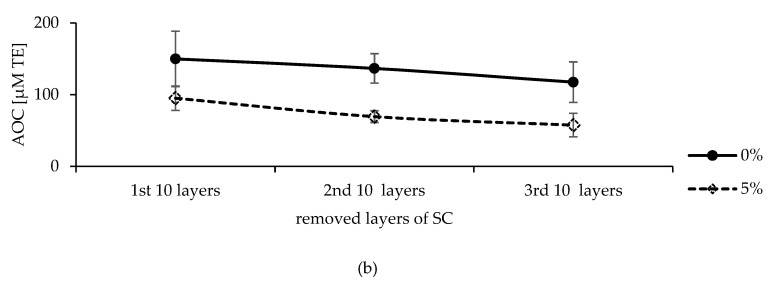
AOC of the removed SC layers after treatment with (**a**) AA solution (20% (w/v)), and (**b**) CoQ10 solution (5% (w/v)), compared to untreated SC layers.

Detailed assessment was performed for further understanding of the results, i.e., the AOC of 10 layers of the SC was analysed separately after treatment with AA and CoQ10 high doses. The AOC values are shown in Figure 10 and revealed different behaviours for each antioxidant compared to the untreated skin. The AOC enhancement that resulted from AA was limited to the upper SC layers in line with its known poor penetration profile. On the contrary, the negative influence of CoQ10 was found to be extended to the deeper layers of SC.

## 3. Discussion

ORAC assay is one of the most used antioxidants assays and has special advantages if the samples contain multiple antioxidants, such as biological samples. Most importantly, it offers a direct measurement of lipophilic and hydrophilic antioxidant capacity [42]. Additionally, ORAC approach is useful for both slow- and fast-reacting antioxidants, as it combines the initial rate and lag time methods [42]. In Figure 2, the long decay times for the skin sample (when compared with Trolox) could be attributed to the complex nature of antioxidants within the skin. For example, α-tocopherol, which is one of the main lipophilic skin antioxidants, is a fast-reacting antioxidant, while glutathione as another essential skin component requires longer reaction times [43]. In this study, ORAC was successfully utilised to indirectly evaluate the iAOC of porcine skin and to determine the iAOC of many variable samples, thus proving its suitability for the intended use.

The intraindividual iAOC study revealed a significantly higher iAOC for skin from the dorsal side of the ears, mostly caused by the higher quantity of removed corneocytes as detected by the IR-D for both sides. The accuracy of the measurements via IR-D showed high creditability based on data from other studies to indirectly estimate the amount of removed corneocytes [27,38,44,45]. The average cumulative protein absorption of the 30 removed layers from all the samples was 282% ± 48% for dorsal sides and 218% ± 43% for ventral sides. Following the literature, these values represent 86% ± 15% and 67% ± 13% of the SC compared to the obtained measurement for the entire SC (IR-D value of 327% ± 133%) [44]. More precise data can be anticipated from IR-D reading via the equation provided by Hoppel et al., which can be used to calculate the thickness of the removed SC to support this finding [46]. Therefore, the estimated removed thickness of the SC by 30 tapes represents 6.9 ± 1.2 and 5.3 ± 1.1 µm of SC on the dorsal and ventral sides, respectively.

The obtained results showed a positive correlation between iAOC and the indirect measurement of the average cumulative protein absorption. In other words, removing more cells implies a higher amount of skin components, more antioxidants removed, and therefore detected, as higher ORAC values. However, the analysis of each dataset for each ear independently revealed a wide correlation range, i.e., ± from 0.3 to 0.7. This variation can be explained by the impact of the tape stripping procedure on the IR-D. Tape stripping removes the porcine corneocytes in an irregular pattern, i.e., it does not consistently remove the skin layer by layer. Thus, the IR-D readings may have been influenced by this irregularity, reflecting occasional imprecise protein absorption [44].

The main explanation for the difference in the amount of removed corneocytes is justified by the difference in skin nature from both sides, meaning that the 30 tapes were able to remove more cells from the dorsal side than from the ventral side. Previous studies have extensively reported on the differences between porcine skin areas, such as the study by Turner et al. [47]. Interestingly, their study found marked histological variations in porcine skin taken from different regions, including the two sides of the ear. The main differences were in the number of cells of various types in each layer, for example, melanocytes and Langerhans cells, and in the thickness of the dermis layer. A higher number of melanocyte cells can be a crucial factor because they have marked AOC values [48]. The dorsal skin from the ears also showed a higher hair density than the skin from all other areas of the body [47]. From a physiological point of view, the ventral side is subject to oily ear discharge (oily secretions). Such secretions may form a film on the skin that prevents the optimal removal of the horny layer. Even though the film will be removed after few strips, the overall amount of the removed SC will be affected. Accordingly, the dorsal sides showed higher iAOC values due to the higher removal of corneocytes via tape stripping and a higher number of melanocytes present.

In the study of ears storage conditions, the stability of antioxidants within the skin after storage in the refrigerator (4 ± 1 °C) is consistent with reported data in the literature for the continued viability of skin cells after storage under similar conditions and periods [49]. The obtained results emphasized that the antioxidants were stable in the skin tissue, since storing of the entire ear did not negatively impact cell integrity or exposed its biochemicals. For example, tocopherol remained localised mainly in the mitochondrion and endoplasmic reticulum inside the cells, while melanin remained localised to its normal storage site, the melanosomes [4]. In a different manner, ascorbic acid was still available cellularly in the mitochondrion and extracellularly, as it has a hydrophilic nature [5].

Regarding the storage at room temperature for 24 h, there are two reasons for the elevation of the iAOC. First, the antioxidant effect of the skin flora must be considered assuming there was a flora sprout during storage, leading to the marked elevation of the AOC. This assumption is supported by reported data that confirm the AOC properties of some skin flora [50]. Additionally, room temperature is a more favourable condition for bacterial growth compared to cold storage [51], and tape stripping is one of the proposed approaches for collecting skin flora for bacterial analysis [52]. The second reason for the elevated iAOC during storage is the production of lactic acid as a part of postmortem changes that occur in the ears about 4–6 h after porcine slaughtering [40]. Lactic acid has been proven to have a concentration-dependent AOC [53], and its production in the skin increases at room temperature due to the increased skin decomposition under such conditions.

In the third part of this research, the antioxidant stability in the tapes were studied because it is a critical issue in the field of antioxidants studies. They are prone to oxidation, photoreaction and hydrolysis. For this reason, the stability of antioxidants in the tapes was explored in different conditions for several time frames. A close review of the literature revealed the usage of a variety of storage conditions in skin antioxidant studies, such as −70 °C in a study by Grazul-Bilska et al. [54], 4 °C for biopsy samples in other studies [31,55] and −20 °C for the separated epidermis, biopsies and excised skin in work by others [56,57,58]. Furthermore, a temperature of −20 °C was found to be effective in the storage of tapes after tape stripping for penetration studies [59].

Overall, the storage was promising when compared to the freshly analysed samples. Storage for up to 48 h successfully preserved the iAOC, which will offer a longer time for sample analysis in prospective studies. Afterwards, the antioxidants began to degrade, and the iAOC declined when analysed after 72 h. The decline was significant for the samples stored at room temperature with *p* = 0.0010. Storage at refrigerator is applicable normally for skin storage [60,61]. However, the storage of the whole skin appeared to be different than storing the skin separate layers in tapes, as the skin lost its integrity and became more exposed and vulnerable.

Further storage at room temperature for up to one week boosted the iAOC, which is in line with the previous findings from the second part of this study. The primary difference, in this case, is that the iAOC elevation was noted after one week, not after 24 h. This difference can be justified by the maintenance of the normal physiology of the skin, which promoted microbial growth in the case of the storage of the whole ears. While in the tapes, each SC layer adhered to a specific tape, the flora growth and the skin decomposition were limited.

Storage in the freezer (−20 ± 2 °C) was found to be a suitable storage protocol for tapes to preserve antioxidants in the long term. It prevented additional reactions of the skin’s enzymatic and nonenzymatic antioxidants for up to eight weeks without a significant decline in the AOC.

The obtained findings in the first two parts of the study ensured the high accuracy of the used model to predict the oxidative changes in porcine skin, as it detected discriminative results for the inter- and intraindividual differences. Moreover, the storage conditions in the third part were optimised, which guarantees more reproducibility and effectiveness in the prospective studies. At that point, it became ready for further applications. Therefore, it was used for the assessment of pro-oxidative and antioxidative agents.

As predicted, the iAOC declined significantly in the skin exposed to UV radiation, and our model had the ability to track the oxidative changes after the exposure. Furthermore, the examination of the exposure time (1 and 2 h) as an influencing factor disclosed distinguishable results. A condition of compromised oxidative state begins even after 1 h of UV radiation, which is a high-risk factor for the development of skin pathologies. This effect resulted mainly from the generation of free radicals upon exposure, such as the reactive oxygen species (ROS), reactive nitrogen species (RNS) and oxygenated lipid species (LOS) [62]. In the long term, the accumulation of these radicals alters the skin elements and initiates irritation, inflammations, degenerative changes and tumours [63]. Thereby, the time-dependent changes due to UV radiation were revealed effectively using this model, which can be a valuable method to assess the impact of all the environmental pro-oxidants on skin.

For the antioxidative effect, two antioxidants were selected because they are highly consumed in skincare cosmetics. Additionally, they are known for their poor permeation into the skin, so they will be retained in SC and their measurement will be possible. The AOC enhancement of AA was expected and concurred well with the other studies that confirmed its cellular and clinically positive influence [64,65]. The acquired linear in vitro AOC response up to 20% was also reflected ex vivo and confirmed by a significant correlation study. In a related dose-perspective study by Pinnell et al., the examination of the skin level of the AA (5–30%) upon the topical application was conducted [66]. Interestingly, they determined the optimal concentration for the maximal percutaneous absorption to be 20% of the drug. After the application of the higher doses (25 and 30%), the AA skin level declined and almost reached 500 pmol/mg for the 30%, which is nearly the level obtained after the use of the 10% formulation. Their findings are in good agreement with ours, and the possible reason for this phenomenon is the saturation of the active and inactive transportation of the molecule into the skin. Active transport is dependent mainly on the sodium-ascorbatecotransporter−1 (SVCT1), which is responsible for the transport of AA in the epidermal tissues [67], while the inactive mechanism involves the simple solvent drag mechanism, as described by Kaushik et al., for the dissolved drugs in liquids [39].

CoQ10 application demonstrated a fairly different pattern, which is dependent on the utilized dose. The lowest dose behaved normally as an antioxidant, while the higher doses acted differently despite their high AOC in the in vitro study. Results emphasized the turn of 5% dose into an aggressive pro-oxidative agent. The clinical observations by Schallreuter et al. supported the direction of our findings, whereas 15 patients showed up with serious cases of isolated facial vitiligo after the use of CoQ10 skincare products [68]. Further investigations were conducted using Fourier transform (FT) Raman spectroscopy, and a confirmation was obtained by a clear conclusion: H_2_O_2_ radicals were generated by the topical application of CoQ10. All these findings raised critical safety concerns regarding the use of this active ingredient in cosmetics, along with the other antioxidants with comparable physicochemical profile. The proposed model in this paper may play a significant role in the safety studies in the premarketing phase, with the advantages of accuracy, ease of use and cost-efficacy.

Although the ex vivo studies are providing the field with a high valued and ethically accepted knowledge, it is worth mentioning that they are not completely resembling the in vivo response because of the lack of oxygen supply chain in the porcine tissues [69,70]. Nevertheless, they are still crucial approaches to be used to understand the primary responses and ensure the initial safety profile before employing the in vivo trials. Future studies that employ the proposed model shall provide more knowledge on the suitability and the ex vivo/in vivo correlation of the model with real world samples.

## 4. Materials and Methods

### 4.1. Tape Stripping

Pig ears were obtained from a local slaughterhouse and immediately rinsed with cold tap water and gently dried with clean paper towels, and the hair was carefully shaved to avoid scratching the surface of the skin. Six defined scratch-free areas of 1.5 × 2 cm^2^ were marked and used for tape stripping. Adhesive tape (Tesafilm^®^ crystal clear, tesa SE, Beiersdorf AG, Norderstedt, Germany) was placed on the skin by applying a defined pressure with a roller (RENOVO, Brillux GmbH & Co. KG, Münster, Germany) [26], followed by rapid removing in one movement with a layer of SC. In the fourth study, tape stripping was performed after the particular skin treatment: UV radiation, CoQ10 and AA application. For all the samples, a total of 30 SC layers of SC were removed from the skin area. The tapes were collected in 3 sets, each containing 10 SC layers, respectively. Each set of tapes was subsequently extracted with 70% (v/v) ethanol in purified water (PURELAB Flex 2, ELGA LabWater, High Wycombe, UK), followed by shaking for 1 h at 160 rpm using an Edmund BühlerSwip KS-10 (Edmund Bühler GmbH, Bodelshausen, Germany). The SC extracts obtained were analysed via ORAC assay.

### 4.2. Skin Treatment

After establishing the ex vivo skin model, several treatments were applied to investigate its potential and extend its applications. To ensure the use of healthy skin, Tewameter^®^ TM 300 and Corneometer^®^ (Courage + Khazaka electronic GmbH, Köln, Germany) were used to measure skin barrier functions and hydration, respectively. Skin with a value lower than 12 g/hm^2^ for the trans epidermal water loss (TEWL) and with a value higher than 20 for hydration was considered as intact skin and thereby used for further investigations.

The initial application was performed using UV radiation as a pro-oxidative agent. The porcine ears were exposed to the radiation of a 250 W UV hand lamp (UV Light Technology Limited, Birmingham, UK) for 1 and 2 h inside a metallic box.

The next experiment was designed to assess the anticipated antioxidative effect. This step was achieved by employing finite doses of 6.3 µL/cm of AA (L-ascorbic acid, Sigma–Aldrich Chemie GmbH, Steinheim, Germany) hydrophilic solutions (1–30% (w/v)) and CoQ10 (Coenzyme Q10, Dr. Rimpler, Wedemark, Germany) (0.5–5% (w/v)) in Miglyol^®^ 812 (Caesar & Loretz GmbH, Hilden, Germany). Solutions and blank solutions were applied and incubated for 2 h at 32 °C. After that, the residuals were wiped off gently by wet and dry paper tissue followed by tape stripping as described earlier.

### 4.3. Quantification of Protein Content

After tape stripping, the absorbance of each tape was measured using an infrared densitometer (IR-D) (SquameScan 850A, Heiland Electronic GmbH, Wetzlar, Germany) to detect the optical pseudo-absorption of the individual tapes at a wavelength of 850 nm. The measured absorbance has a linear correlation with the protein content in the tapes [44,45]. This is a simple and nondestructive method for quantifying the amount of skin corneocytes [27,44]. The results were expressed as an average cumulative protein absorption (%).

### 4.4. Storage and Extraction of Tapes

The tapes were placed in tightly secured glass vials and stored according to the specifications of the study outline. In the first and the second part, the tapes were stored in the refrigerator and analysed within 24 h, whereas in the third part of the study, tapes were stored at room temperature (20 ± 2 °C), in the refrigerator (4 ± 1 °C) and in the freezer (−20 ± 2 °C) to investigate their short- and long-term stability. At last, depending on the findings of the third part, the tapes of the fourth part were stored directly at −20 ± 2 °C and analysed within 10 days.

Each set of tapes was quantitively extracted with 70% ethanol in purified water (PURELAB Flex 2, ELGA LabWater, High Wycombe, UK), followed by shaking for 1 h at 160 rpm using an Edmund BühlerSwip KS-10 (Edmund Bühler GmbH, Bodelshausen, Germany), and analysed via ORAC assay.

### 4.5. ORAC Assay

**Figure 11:**
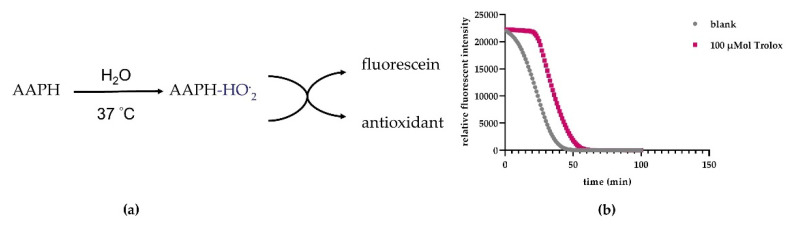
(**a**) Primary reaction of ORAC assay and (**b**) time-dependent fluorescence decay curve with and without antioxidant (Trolox).

The modified ORAC assay described by Ou et al. was adopted from the food chemistry literature and applied to the skin [41,71,72]. The primary reaction in ORAC between fluorescein and 2,20-azobis(2-methylpropionamidine) dihydrochloride (AAPH) as a free radical decreases the relative fluorescence intensity of fluorescein in a time-dependent decay curve Figure 11. The presence of antioxidants (e.g., Trolox) in the medium slows this reaction competing with the fluorescein. Consequently, the decay curve is delayed.

The assay procedure can be summarised as follows: Trolox (6-Hydroxy-2,5,7,8-tetramethylchroman-2-carboxylic acid, Santa Cruz Biotechnology Inc., Dallas, TX, USA) was used as an external standard, AAPH (Acros Organics, Geel, Belgium) served as the source of free radicals and fluorescein (Alfa Aesar, ThermoFisher GmbH, Kandel, Germany) was utilized as the fluorescent probe. Trolox standards were prepared in concentrations of 22.2 to 100 µM, AAPH in a concentration of 125 mM and fluorescein in 1 µM. All chemicals were prepared in a physiological buffer (pH = 7.4) to simulate normal physiology. Of each standard/sample, 20 µL were placed in a black 96-well plate, and 150 µL of fluorescein solution was added, followed by incubation at 37 °C for 10 min. Freshly prepared AAPH solution was then combined to each well in a volume of 90 µL. After that, the plate was inserted immediately into the plate reader (FluoStar^®^ Optima plate, BMG Labtech, Offenburg, Germany), and the measurement began with excitation of 485 nm and emission of 520 nm for 100 min. The area under the curve (AUC) of each standard/sample was calculated by plotting the relative fluorescence intensity versus time. Finally, the calibration curve was obtained using the concentration of Trolox standards and their AUC. The fluorescence intensity of each sample, transformed into their AUC, was used to get the AOC values. The regression equation of the calibration curve was applied, and the Trolox concentration that correspond to the samples AUC was expressed as µM Trolox equivalents (µM TE).

### 4.6. Statistics

Excel (Microsoft 365) was used for graphs and descriptive analysis, and GraphPad Prism (version 9–9.3.1, GraphPad Software, San Diego, CA, USA) was used for ORAC calculation and statistical analysis. Each dataset was tested for normality, and then t-test or ANOVA and post hoc analysis (Tukey and Dunnett’s) were applied for comparison. Pearson’s r was the correlation analysis between IR-D measurements and their corresponding iAOC. Experiments were performed in triplicate, results were expressed as mean ± standard deviation (SD) and *p* values < 0.05 were considered statistically significant.

### 4.7. Normalisation

For a better comparison of iAOC data from different spots of the ear, a normalization of the data was performed. Based on the significant correlation between IR-D measurements and their corresponding iAOC, the data were normalised according to the following equation:

$$normalised\;\mathrm{iAOC}\;\left[\text{µ}M\;TE\right]=\frac{\mathrm{iAOC}\;\left[\text{µ}M\;TE\right]}{average\;cumulative\;protein\;absorption\;\left(\%\right)}\times 100$$

## 5. Conclusions

The results of the obtained study demonstrated that the combination of classical tape stripping and ORAC assay is a suitable tool that allows one to evaluate the antioxidant state of the skin. A baseline of the iAOC was determined for intact, untreated skin. Accordingly, variations in the iAOC become distinguishable via the presented method and lower values—caused by various treatments—might be considered as a risk factor for skin irritations, promoted skin aging, autoimmune diseases or cancer.

Furthermore, the effect of different storage conditions was defined, and several factors were found to impact the iAOC. They include the ears’ storage at room temperature and the tape storage at refrigerator and room temperature. Therefore, the optimum conditions that reserve the iAOC were standardised and can be the guidelines for future research using this method.

Finally, the proof-of-concept study (skin treatment with UV, AA, CoQ10) could confirm the sensitivity of the model, i.e., its ability to detect oxidative changes in skin upon the exposure to pro-oxidative and/or antioxidative agents. Hence, the ex vivo model can be considered to be a valuable approach for many applications, i.e., many applications can be accomplished to inspect the potential and the safety of cosmeceutical formulations.

Future studies that extend the applications and the use of the model—not only ex vivo but also for in vivo applications—are now needed to further validate and improve the presented ex vivo iAOC skin model.

## Data Availability

Data is contained within the article.
